# The Autophagy Cargo Receptor SQSTM1 Inhibits Infectious Bursal Disease Virus Infection through Selective Autophagic Degradation of Double-Stranded Viral RNA

**DOI:** 10.3390/v13122494

**Published:** 2021-12-13

**Authors:** Chenyang Xu, Tongtong Li, Jing Lei, Yina Zhang, Jiyong Zhou, Boli Hu

**Affiliations:** 1 MOE International Joint Collaborative Research Laboratory for Animal Health and Food Safety, College of Veterinary Medicine, Nanjing Agricultural University, Nanjing 210095, China; 2016207012@stu.njau.edu.cn (C.X.); 2018107083@njau.edu.cn (T.L.); leijing@njau.edu.cn (J.L.); 2 MOA Key Laboratory of Animal Virology, Center for Veterinary Sciences, Zhejiang University, Hangzhou 310058, China; 88173611zyn@zju.edu.cn (Y.Z.); jyzhou@zju.edu.cn (J.Z.)

**Keywords:** infectious bursal disease virus (IBDV), dsRNA, autophagy, SQSTM1, VPS34

## Abstract

Selective autophagy mediates the degradation of cytoplasmic cargos, such as damaged organelles, invading pathogens, and protein aggregates. However, whether it targets double-stranded RNA (dsRNA) of intracellular pathogens is still largely unknown. Here, we show that selective autophagy regulates the degradation of the infectious bursal disease virus (IBDV) dsRNA genome. The amount of dsRNA decreased greatly in cells that overexpressed the autophagy-required protein VPS34 or autophagy cargo receptor SQSTM1, while it increased significantly in *SQSTM1* or *VPS34* knockout cells or by treating wild-type cells with the autophagy inhibitor chloroquine or wortmannin. Confocal microscopy and structured illumination microscopy showed SQSTM1 colocalized with dsRNA during IBDV infection. A pull-down assay further confirmed the direct binding of SQSTM1 to dsRNA through amino acid sites R139 and K141. Overexpression of SQSTM1 inhibited the replication of IBDV, while knockout of *SQSTM1* promoted IBDV replication. Therefore, our findings reveal the role of SQSTM1 in clearing viral dsRNA through selective autophagy, highlighting the antiviral role of autophagy in the removal of the viral genome.

## 1. Introduction

Autophagy mediates the clearance of damaged or redundant cellular components, such as protein aggregates, damaged organelles, or invading pathogens. Initiation of autophagy is mediated by the VPS34-Beclin-1 complex and the MTOR signaling pathway [1]. Autophagy can be either nonselective or selective. Nonselective autophagy is a cellular response to nutritional deficiencies, in which the cytoplasm is nonspecifically engulfed into autophagosomes, while selective autophagy is responsible for removing components specifically through cargo receptor-mediated recognition [2,3]. SQSTM1 is one of the most well-known cargo receptors and plays an important role in recognizing and removing aggregates, mitochondria, and pathogens [4]. In addition, SQSTM1 was found to interact with a vault RNA directly, and the interaction prevented the oligomerization of SQSTM1 and subsequent selective autophagy [5]. However, whether SQSTM1 facilitates the degradation of dsRNA is still unknown.

The RNA metabolism involved in RNA synthesis, processing, folding, modification, and degradation ensures proper RNA functions. RNA molecules are monitored for quality control. Faulty and excessive RNA molecules are eliminated by RNA decay enzymes [6,7], such as ribonuclease, RNA helicase, and other RNA binding proteins. However, a portion of cellular components, such as rRNA and tRNA, are not accessible to the RNA decay enzymes due to their highly structured and extensively restricted localization within ribonucleoprotein complexes and are difficult to degrade by canonical RNA degradation mechanisms. Autophagy is effective at removing these types of RNA [8]. For example, cytoplasmic RNA granules, retrotransposons [9], RNA-protein aggregates, and viral RNA could be degraded through autophagy [10,11]. However, whether long dsRNA from invading pathogens is subjected to autophagic degradation is largely unknown.

IBDV is a nonenveloped dsRNA virus that causes immunosuppressive and highly contagious diseases in young chickens. IBDV contains two bisegmented double-stranded RNA genomes called segments A (3.2 kb) and B (2.8 kb) [12]. Genomic dsRNA binds to the cellular pattern recognition receptor MDA5 to initiate type I interferon (IFN) production [13]. However, whether and how autophagy recognizes and removes cytoplasmic IBDV dsRNA is still unclear.

Here, our study shows that SQSTM1 directly interacts with IBDV dsRNA and mediates its degradation through autophagy. Our study provides evidence that the pathogen genome could be removed by SQSTM1-mediated selective autophagy, highlighting the antiviral role of autophagy during virus infection.

## 2. Materials and Methods

### 2.1. Cells, Virus, and Reagents

HEK293T cells (CRL-11268, ATCC, Rockefeller, MD, USA) and the chicken fibroblast cell line DF-1 (CRL-12203, ATCC, Rockefeller, MD, USA) were routinely grown in Dulbecco’s modified Eagle’s medium (DMEM; Gibco, Carlsbad, CA, USA) supplemented with 10% fetal bovine serum (CCS30010.02; MRC, Lytton, QLD, Australia). IBDV strain NB (NB virus) isolated by the Key Laboratory of Animal Virology (Hangzhou, China) was adapted to growth in DF-1 cells [14]. *VPS34* KO cell lines were derived from the MOA Key Laboratory of Animal Virology (Hangzhou, China) [15]. *SQSTM1* KO cell lines were stored in the MOA Key Laboratory of Animal Virology (Hangzhou, China).

### 2.2. Antibodies

Rabbit polyclonal antibody against GAPDH (glyceraldehyde-3-phosphate dehydrogenase; ABPR001) was purchased from Xianzhi Biological Technology (Hangzhou, China), and antibodies against Myc (R1208-1) and GST (EM80701) were purchased from Huaan Biological Technology (Hangzhou, China). Anti-SQSTM1 antibody (ab109012) was purchased from Abcam (Cambridge, UK). Mouse monoclonal antibodies to the viral protein VP2 of IBDV were provided by the Key Laboratory of Animal Virology [16,17]. Horseradish peroxidase (HRP)-conjugated anti-mouse (074-1806) and anti-rabbit IgG (074-1506) were obtained from KPL (Milford, MA, USA). Alexa Fluor 546-conjugated anti-rabbit (A21085) and anti-mouse IgG (A10036) were purchased from Invitrogen (Carlsbad, CA, USA). Fluorescein isothiocyanate (FITC)-conjugated anti-mouse (172-1806) and anti-rabbit IgG (172-1506) were purchased from KPL (Milford, MA, USA). NP-40 lysis buffer (50 mM Tris [pH 7.4], 150 mM NaCl, 1% NP-40; P0013F) was purchased from Beyotime (Shanghai, China). Anti-GST resin (L00206) was obtained from Genescript (Nanjing, China).

### 2.3. Plasmids and Transfection

The full-length SQSTM1 gene was amplified by PCR from the cDNA of 293T cells and inserted into the plasmids pCMV-Myc-N (Clontech, Mountain View, CA, USA), pGEX-4T-1 (GE, Boston, MA, USA), and pCDH-CMV-MCS-EF1-Puro (SBI, Palo Alto, CA, USA), thereby designated as Myc-SQSTM1, GST-SQSTM1, and pCDH-SQSTM1, respectively. All constructs were confirmed by sequencing (Zhejiang Sunya Biotechnology Co, Hangzhou, China). All plasmids and RNA were transfected into cells using lipofectamine 3000 reagent (Invitrogen, Carlsbad, CA, USA) according to the manufacturer’s instructions.

### 2.4. Construction of SQSTM1 Knockout Cell Lines

293T cells were cultured in DMEM supplemented with 10% fetal bovine serum. The gRNA was designed online (http:crispr.mit.edu/ accessed on 18 June 2018), the sgRNA sequence against SQSTM1 (sense: TAACTTACCATAGACATCTG antisense: CAGATGTCTATGGTAAGTTA) was inserted into the CRISPR/Cas9 plasmid PX459, which contains puromycin resistance. The reconstructed plasmids were transfected into 293T cells and then screened with puromycin at a concentration of 2 µg/mL. The survived cells were diluted into a 96-well plate to screen monoclonal cells. After that, knockout effects were validated by Western blot and sequence. The sequencing result shows the amplified fragment that covered the deletion region was totally removed.

### 2.5. Construction of STable 293T Cells Expressing SQSTM1

PCDH-SQSTM1 was cotransfected with the ViraPower^TM^ lentiviral packaging mix (K497500; Invitrogen, Carlsbad, CA, USA) into 293T cells to generate a lentiviral stock according to the manufacturer’s protocol, and an empty vector was used as control. After 72 h of transfection, viral particles were acquired from the medium by ultracentrifugation. After lentiviral preparation, 293T cells were seeded in the 6-wells plate and grown to 80% confluence overnight. 293T cells were separately infected with 2 mL pCDH-SQSTM1 or pCDH-CMV-MCS-EF1 virus for 6 h. After cells were cultured for another 24 h with complete medium, puromycin (4 µg/mL) was added to screen positive cells. Western blot and indirect immunofluorescence assay were used to detect the expression of SQSTM1 in the cell lines.

### 2.6. Cytotoxicity Assay

The cytotoxicity assay was performed using an enhanced CCK8 kit (C0014, Beyotime, Shanghai, China) according to the standard protocol. The cells seeded in 96-well plates were treated with the drug for 4 h or transfected with vectors for 8 h. After treatments or transfection, 10 µL CCK-8 reagent was added into the culture medium. After incubation for 1 h, the absorbance value was measured at 450 nm with a spectrophotometer. The cell viability was calculated as (A450_treated_/A450_control_).

### 2.7. IBDV Genomic dsRNA Extraction

DF-1 cells reached about 80% confluence, and culture medium was replaced with medium containing 2% FBS. The cells were infected with IBDV (MOI = 0.1) and cultured for 48 h. Then, the cells were frozen and thawed 3 times and centrifuged for 10 min. The supernatant was transferred to clean tubes and centrifuged at 50,000× *g* for 4 h. The pellet was resuspended in PBS (1% of the medium volume), and then 10% SDS (5% of the PBS volume) and proteinase K (1 mg/mL) were added to digest the viral proteins at 50 °C for 2 h. The dsRNA was purified by phenol/chloroform/isoamyl alcohol (25:24:1) extraction and precipitated with isopropanol. Finally, the dsRNA was dissolved in diethyl pyrocarbonate (DEPC)-treated water. Purified dsRNA was separated in 1% agarose gel and detected by a gel imaging analysis system (GenoSens 1880, Clinx, Shanghai, China).

### 2.8. IBDV dsRNA Degradation Assay

The dsRNA of IBDV was transfected into wild-type, *VPS34* KO or *SQSTM1* KO 293T cells at approximately 80% confluence by using lipofectamine 3000 (L3000015, Invitrogen, Carlsbad, CA, USA). The cells were harvested at 8 h post transfection. Then, the cells were lysed with Trizol, and total RNA was extracted with chloroform. Reverse transcription of total RNA was carried out using a first-strand synthesis system, and cDNA was obtained. The amount of dsRNA was measured by qRT-PCR and PCR.

### 2.9. Detection of IFN-β and IFN-Stimulated Gene

Myc-SQSTM1 or its variant Myc-SQSTM1^RK/A^ and dsRNA were cotransfected into *SQSTM1* KO cell lines. The cells were harvested at 8 h post transfection. Then cells were lysed with Trizol, and total RNA was extracted with chloroform. Reverse transcription of total RNA and cDNA was obtained. The relative levels of IFN-β mRNA, IFN-stimulated gene mRNA, and β-Actin mRNA from *SQSTM1* KO cell lines were analyzed by qRT-PCR.

### 2.10. Primer Pairs for qRT-PCR and PCR

Primer pairs were as follows: for dsRNA (sense: CCTCTGGGAGTCACGAATTAAC; antisense: ACTCATGGTGGCAGAATCATC), for β-actin in 293T cell lines (sense: TCTGGCACCACACCTTCTAC, antisense: ATCTGGGTCATCTTCTCGC) and GAPDH in DF-1 cell lines (sense: CCCAGCAACATCAAATGGGCAGAT, antisense: TGATAACACGCTTAGCACCACCCT), for IFN-β in 293T cell lines (sense: TTGTTGAGAACCTCCTGGCT, antisense: TGACTATGGTCCAGGCACAG), for ISG56 in 293T cell lines (sense: TCATCAGGTCAAGGATAGTC, antisense: CCACACTGTATTTGGTGTCTAGG), for ISG15 in 293T cell lines (sense: AGGACAGGGTCCCCCTTGCC, antisense: CCTCCAGCCCGCTCACTTGC), for dsRNA analog 1 (sense: ACTACCAGCAGAACACCCCCATCGG, antisense: GCAGGACCATGTGATCGCGCTTCTC), for dsRNA analog 2 (sense: GAATCAGGGGATAACGCAGGAAA, antisense: GTAAGCGGCAGGGTCGGAACA), for IBDV A segment (sense: CACCAGAATGGGTAGCA, antisense: ATCGCAGTCAAGAGCAGA), for IBDV segment B (sense: CCTCTGGGAGTCACGAATTAAC; antisense: ACTCATGGTGGCAGAATCATC).

### 2.11. Indirect Immunofluorescence Assay

To observe the transfection efficiency of SQSTM1 and VPS34, 293T cells were transfected with Myc-SQSTM1 or Myc-VPS34 for 8 h, while DF-1 cells were transfected with Myc-chSQSTM1 for 8 h. Then, the cells were fixed with 4% paraformaldehyde for 30 min and permeabilized with 0.2% Triton X-100 for 10 min. Cells were incubated with an anti-Myc-tag antibody at 37 °C for 2 h. After three washes with PBST, cells were incubated with FITC-conjugated goat anti-rabbit IgG at 37 °C for 1 h. After three washes with PBST, the cells were incubated with 4′,6-diamidino-2-phenylindole (DAPI) to stain the nuclei. Then, the cells were observed under a fluorescence microscope (Ti-E, Nikon, Minato-ku, Tokyo, Japan).

### 2.12. Confocal Microscopy and Structured Illumination Microscopy

To observe the colocalization of IBDV dsRNA and SQSTM1, DF-1 cells were transfected with Myc-SQSTM1 for 24 h and then infected with IBDV (MOI = 0.1) for 24 h. The cells were then fixed with 4% paraformaldehyde for 30 min and then permeabilized with 0.2% Triton X-100 for 10 min. The fixed cells were incubated with mouse anti-dsRNA monoclonal antibody and rabbit anti-Myc monoclonal at 37 °C for 2 h. After three washes with PBST, the cells were incubated with FITC-conjugated goat anti-rabbit IgG- and A546-conjugated donkey anti-mouse IgG at 37 °C for 1 h. After three washes with PBST, the cells were incubated with DAPI. The cells were observed under a Nikon laser confocal microscope (Ti-E + A1, Nikon, Minato-ku, Tokyo, Japan) and N-SIM microscope (Ti-E + SIM, Nikon, Minato-ku, Tokyo, Japan).

### 2.13. Western Blot

The cells were resuspended in PBS and centrifuged at 1000× *g* for 10 min at 4 °C. The cell pellets were lysed in NP-40 buffer. Then, SDS-PAGE loading buffer (P1016, Solarbio, Beijing, China) was added to the cell lysates to prepare the samples. Equivalent amounts of samples were subjected to SDS-PAGE, transferred to nitrocellulose membranes, and then incubated with 5% skimmed milk dissolved well in PBST at 37 °C for 40 min. After three washes with PBST, the membrane was incubated with primary antibodies at 4 °C for 8 h. After four washes with PBST, the membrane was incubated with HRP-conjugated anti-mouse or anti-rabbit IgG at 37 °C for 40 min. After three washes with PBST, the membrane was visualized using a SuperSignal West Femto Maximum Sensitivity Substrate (34094, Thermo Fisher Scientific, Waltham, MA, USA) and imaged using a chemiluminescence imaging system(GE Amersham Imager680, Boston, MA, USA). In addition, densitometric analysis was performed.

### 2.14. RNA Binding Protein Immunoprecipitation Assay

Purified IBDV dsRNA was incubated with anti-GST resin at 4 °C for 1 h, and the dsRNA with nonspecific binding beads were removed by centrifugation at 1000× *g* for 5 min. Then, the supernatant was mixed with prokaryotically expressed recombinant GST-SQSTM1 protein and rotated at 4 °C for 2 h. The anti-GST resin was then added to the mixture and rotated at 4 °C for 2 h. After four washes with NP-40 lysis buffer, the samples were divided into two parts. One part was used to detect protein expression by Western blot; the other part was used to detect the bound RNA by PCR.

### 2.15. RNA Pull-Down

The IBDV genomic dsRNA extraction assay was conducted using a Pierce™ Magnetic RNA-Protein Pull-Down Kit (20164, Thermo Fisher Scientific, Waltham, MA, USA) according to standard protocols. Firstly, the IBDV dsRNA was labeled by using the Thermo Scientific Pierce RNA 3′ Desthiobiotinylation Kit, and then the labeled RNA was bound to streptavidin magnetic beads. After that, GST-SQSTM1 protein was added to the mixture. After two washes with wash buffer, the targeted protein was eluted with elution buffer. The samples were processed and detected by Western blot.

### 2.16. DsRNA Transcription

Both strands of 1000 bp DNA segments from pEGFP-C3 vector (primer pairs for dsRNA analog 1: sense: TAATACGACTCACTATAGGGTCCTACTTGGCAGTACATCT, antisense: TAATACGACTCACTATAGGGGTCCATGCCGAGAGTGATCC), 2810 bp DNA segments from pGL3-Basic vector (primer pairs for dsRNA analog 2: sense: TAATACGACTCACTATAGGGGGTACCGAGCTCTTACGCGT, antisense: TAATACGACTCACTATAGGGTTTTCCGAAGGTAACTGGCT), and DNAs encoding IBDV A and B segments (primer pairs for IBDV A: sense: TAATACGACTCACTATAGGGGGATACGATCGGTCTGACCC, antisense: TAATACGACTCACTATAGGGGGGGACCCGCGAACGGATCC, primer pairs for IBDV B: sense: TAATACGACTCACTATAGGGGGATACGATGGGTCTGACCC, antisense: TAATACGACTCACTATAGGGGGGGGCCCCCGCAGGCGAAG) were transcribed by T7 High-Yield RNA Transcription Kit (TR102, Vazyme, Nanjing, China). Transcriptional RNA was annealed to form double strands. RNase T1 was used to digest single-stranded RNA. DsRNA analogs were separated in 1% agarose gel and detected by a gel imaging analysis system (GenoSens 1880, Clinx, Shanghai, China).

### 2.17. Statistical Analysis

The protein bands were relatively quantified from Western blot analysis. Briefly, the mean gray value of protein bands within the linear range and background was measured by using Image J software (National Institutes of Health, Bethesda, MD, USA), and the quantification reflects the relative amounts as a ratio of each net band value relative to the net loading control. Data statistical significance was calculated by Student’s t-test. All data from three independent biological experiments are presented as mean ± SD (*, *p* < 0.05; **, *p* < 0.01).

## 3. Results

### 3.1. Autophagy Mediates the Degradation of IBDV dsRNA

**Figure 1:**
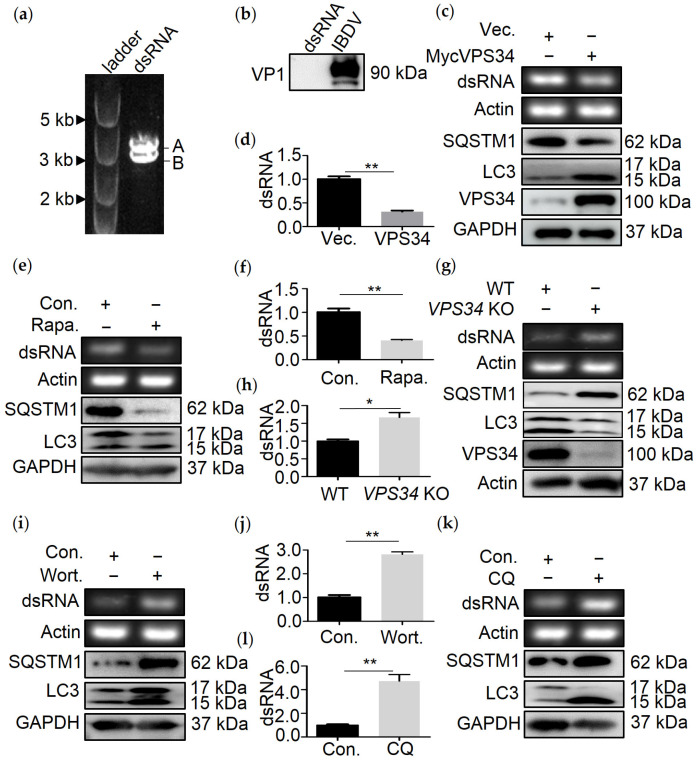
Autophagy promotes the degradation of IBDV dsRNA. (**a**) IBDV dsRNA was extracted from IBDV and separated by agarose gel electrophoresis. DsRNA consists of two bands, segment A (3.2 kb) and segment B (2.8 kb). (**b**) Extracted dsRNA or lysate of virus-infected cells was immunoblotted by using indicated antibody. QRT-PCR and PCR analysis of IBDV dsRNA and β-Actin mRNA from cells cotransfected with Myc-tag human VPS34 (VPS34) and dsRNA for 8 h (**c**,**d**), transfected with dsRNA for 8 h and treated with Rapa (5 µM) for 4 h before collection (**e**,**f**), transfected with dsRNA for 8 h in knock out VPS34 (VPS34 KO) cell lines (**g**,**h**), transfected with dsRNA for 8 h and treated with Wort (20 nM) (**i**,**j**) or CQ (100 µM) (**k**,**l**) for 4 h before collection. Data were represented as mean ± SD of three independent experiments. NS, not significant (*p* > 0.05); * *p* < 0.05, ** *p* < 0.01.

RNA is a substrate of autophagy [8]. However, whether IBDV genomic dsRNA is degraded by autophagy is still unknown. In order to explore this question, IBDV dsRNA was extracted from purified IBDV particles (Figure 1a). Given that viral genomic dsRNA is covalently linked to IBDV VP1 within the virion [18], separation of dsRNA from VP1 was confirmed by Western blot, which showed that extracted dsRNA did not contain VP1 (Figure 1b). We next determined whether dsRNA is degraded through autophagy. 293T cells were transfected with a vector overexpressing VPS34, a kinase that is necessary for inducing autophagy in mammalian cells [19] and was expected to induce autophagy when overexpressed. We, therefore, transfected 293T cells with a vector expressing VPS34. The transfection efficiency of VPS34 in 293T cells was 54% (Figure S1a). Cell viability was no different in cells overexpressing VPS34 than in wild-type (WT) controls (Figure S1b). However, in comparison to WT cells, the level of LC3-II increased in cells expressing VPS34 and treated either with or without CQ, confirming that autophagy was enhanced upon expressing VPS34 (Figure S1c,d). The level of dsRNA was tested in VPS34 expressing cell lines by real-time PCR (qRT-PCR) and PCR. The results showed that amount of dsRNA decreased greatly in VPS34 expressing cell lines compared with WT cells (Figure 1c,d). Moreover, pharmacological induction of autophagy was performed to test the turnover of dsRNA. Treatment with rapamycin (Rapa), an autophagy stimulator, had no effect on cell viability (Figure S1e), resulted in an increase in LC3-I to LC3-II conversion, decreased SQSTM1 levels (Figure 1e), and led to a reduction in dsRNA (Figure 1e,f).

Reversely, turnover of dsRNA was tested upon blocking autophagy by knocking out VPS34 or pharmacological inhibition, such as wortmannin(Wort) or chloroquine (CQ) treatment. Firstly, the results showed that VPS34 knockout (Figure S1f), Wort (Figure S1g), or CQ (Figure S1h) treatment had no effect on the activity of the cells. We next tested the turnover of dsRNA in cells knocked out VPS34 or treated with Wort or CQ. Interestingly, the results showed that the amount of dsRNA increased greatly once autophagy was inhibited by knocking out VPS34 (Figure 1g,h) or treating cells with Wort (Figure 1i,j) or CQ (Figure 1k,l).

Altogether, the above data suggested that autophagy regulates the degradation of dsRNA.

### 3.2. SQSTM1 Is Responsible for the Degradation of dsRNA

**Figure 2:**
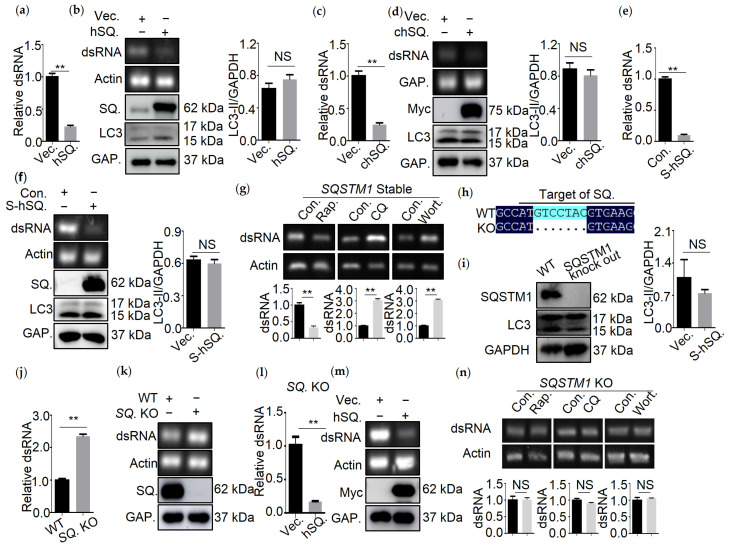
QRT-PCR and PCR analysis of IBDV dsRNA and β-Actin mRNA from 293T cells cotransfected with Myc-tag human SQSTM1 (hSQ) and dsRNA for 8 h (**a**,**b**), DF-1 cells cotransfected with Myc-tag chicken SQSTM1 (chSQ), and dsRNA for 8 h (**c**,**d**), sTable 293T cells expressing SQSTM1 (S-hSQ) transfected with dsRNA for 8 h (**e**,**f**), sTable 293T cells expressing SQSTM1 (SQSTM1 Stable) transfected with dsRNA for 8 h and treated with Rapa., CQ, Wort. for 4 h before collection (**g**). (**h**) Sequencing analysis of DNA amplified from SQSTM1 genes from SQSTM1 KO cell lines. (**i**) Cell lysates from wild-type (WT) or SQSTM1 KO cell lines were subjected to immunoblotting analysis by using indicated antibodies. QRT-PCR and PCR analysis of IBDV dsRNA and β-Actin mRNA from SQSTM1 knock out (SQ. KO) 293T cells transfected with dsRNA for 8 h (**j**,**k**), SQSTM1 KO cell lines cotransfected with Myc-tag human SQSTM1 (hSQ.) and dsRNA for 8 h (**l**,**m**), SQSTM1 knock out (SQSTM1 KO) 293T cells transfected with dsRNA for 8 h and treated with Rapa, CQ, Wort for 4 h before collection (**n**). Data were represented as mean ± SD of three independent experiments. NS, not significant (*p* > 0.05); ** *p* < 0.01.

SQSTM1 is a receptor for selective autophagy, and several reports have demonstrated SQSTM1-mediated degradation of virus components through autophagy [4]. To determine whether the viral genome could be degraded by SQSTM1-mediated autophagy, we measured the amount of IBDV dsRNA in the presence or absence of SQSTM1 expression. Firstly, the transfection efficiency and viability of 293T or DF-1 cells transfected with human SQSTM1 or chicken chSQSTM1 and the viability of sTable 293T cell lines expressing SQSTM1 was determined. The results showed that the transfection efficiency of SQSTM1 or chSQSTM1 was around 66.7% (Figure S1i) or 60.0% (Figure S1k), respectively, and the expression efficiency of SQSTM1 in sTable 293T cell lines was about 83.0% (Figure S1m). The expression of SQSTM1 or chSQSTM1 had no effect on the viability of 293T or DF-1 cells (Figure S1j,l,n). Next, we examined the effects of SQSTM1 or chSQSTM1 expression on dsRNA turnover in 293T or DF-1 cells. As shown in Figure 2a–f, in comparison to empty vector-transfected cells, SQSTM1 or chSQSTM1 expression led to decreased levels of dsRNA as determined by qRT-PCR or PCR analysis, but did not result in changes in LC3-I to LC3-II conversion as determined by Western blot. Consistently, Rapa treatment resulted in a significant decrease in dsRNA, while CQ and Wort treatment led to greatly increased dsRNA in SQSTM1 overexpressing cell lines (Figure 2g).

Next, we generated *SQSTM1* knocked out 293T cell lines through CRISPR/Cas9. The sequencing results showed that seven nucleotides of SQSTM1 genes were deleted (Figure 2h). The Western blot analysis of SQSTM1 confirmed that *SQSTM1* was indeed knocked out (Figure 2i). The proliferation analysis showed that *SQSTM1* KO had no effect on cells viability (Figure S1o). Then, qRT-PCR and PCR analysis of dsRNA confirmed that knocking out SQSTM1 increased the amount of dsRNA (Figure 2j,k), while re-expression of SQSTM1 rescued the turnover of dsRNA in *SQSTM1*-KO cells (Figure 2l,m). Consistently, knocking out of SQSTM1 compromised the effect of Rapa or CQ and Wort treatment on the degradation of dsRNA, suggesting that SQSTM1 was indeed required for autophagic degradation of dsRNA (Figure 2n).

Altogether, SQSTM1 mediates the turnover of dsRNA.

### 3.3. SQSTM1 Binds to dsRNA Directly

**Figure 3:**
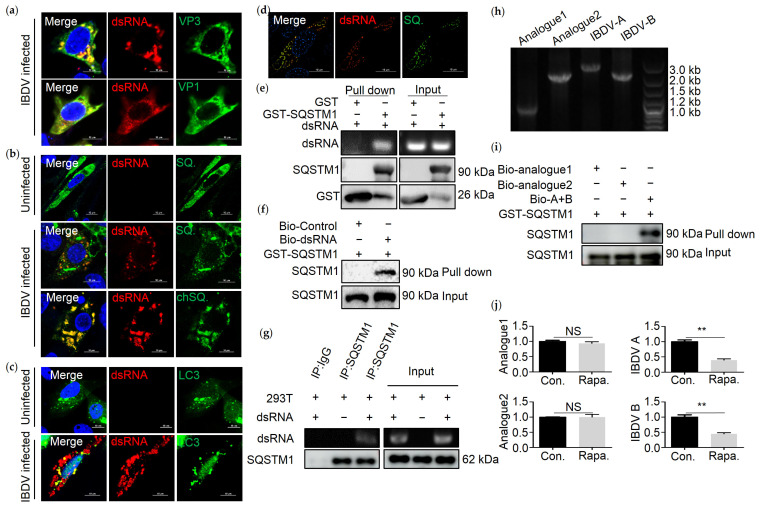
SQSTM1 interacts with dsRNA. (**a**) DF-1 cells were infected with IBDV at MOI = 0.1 for 18 h, followed by confocal analysis of DF-1 cells infected with IBDV by using anti-dsRNA, -VP3, -VP1 antibodies. Scale bar = 10 µm. (**b**) DF-1 cells transfected with Myc-human SQSTM1 (SQ.) or Myc-chicken SQSTM1 (chSQ.) for 18 h, and infected with IBDV at MOI = 0.1 for 18 h (uninfected sample used as control), followed by confocal analysis of DF-1 cells infected with IBDV by using anti-dsRNA and -Myc antibodies. Scale bar = 10 µm. (**c**) DF-1 cells transfected with GFP-LC3 (LC3) for 18 h and infected with IBDV at MOI = 0.1 for 18 h (uninfected sample used as control), followed by confocal analysis of DF-1 cells infected with IBDV by using anti-dsRNA antibodies. Scale bar = 10 µm. (**d**) DF-1 cells transfected with Myc-SQSTM1 for 18 h and infected with IBDV at MOI = 0.1 for 18 h, followed by SIM analysis using anti-dsRNA and -Myc antibodies. Scale bar = 10 µm. (**e**) Prokaryotically expressed protein GST-SQSTM1 binds to IBDV dsRNA. GST-SQSTM1 was incubated with IBDV dsRNA for 4 h, and an RNA binding protein immunoprecipitation assay was performed using anti-GST resin. Purified IBDV dsRNA was pulled down by the GST-SQSTM1 protein but not by the GST tag protein. (**f**) GST pull-down shows that SQSTM1 directly interacts with dsRNA. IBDV dsRNA was labeled with biotin by using a kit. Only the purified IBDV dsRNA can bind to SQSTM1 directly. An RNA pull-down assay was conducted to confirm the interaction between SQSTM1 and dsRNA. (**g**) Endogenous SQSTM1 binds to IBDV dsRNA. 293T cells were incubated with IBDV dsRNA for 4 h. Anti-SQSTM1 precipitation was performed, and IBDV dsRNA was precipitated by endogenous SQSTM1. (**h**) DsRNA nucleic acid analogs were separated by agarose gel electrophoresis, including analog 1(1 kb), analog 2(2.8 kb), IBDV A(3.2 kb), and IBDV B(2.8 kb). (**i**) Prokaryotically expressed protein GST-SQSTM1 interacts with IBDV A and B but not any other dsRNA analogs. DsRNA analogs were labeled with biotin, and a pull-down assay was performed. An immunoblotting analysis using anti-SQSTM1 antibodies was performed. (**j**) QRT-PCR analysis of dsRNA analogs from 293T cells transfected with dsRNA analogs for 8 h and treated with Rapa (5 µM) for 4 h before collection. Data were represented as mean ± SD of three independent experiments. NS, not significant (*p* > 0.05); ** *p* < 0.01.

Since SQSTM1 mediated the degradation of IBDV dsRNA, and a study shows that SQSTM1 binds to vault RNA directly [5], we next tested whether SQSTM1 could bind to dsRNA of IBDV. Confocal and structured illumination microscopy (SIM) analysis of colocalization between dsRNA and SQSTM1 was conducted. In order to validate whether anti-dsRNA antibodies could be used to indicate the IBDV genome, confocal analysis of colocalization between VP1 or VP3 and dsRNA in DF-1 cells infected with IBDV by indirect immunofluorescence assay (IFA) using anti-VP1 or -VP3, and -dsRNA antibodies were conducted. The results showed that dsRNA colocalized with VP1 or VP3 significantly (Figure 3a), suggesting that anti-dsRNA antibodies can be used to label dsRNA from IBDV. Following, DF-1 cells were transfected with Myc-SQSTM1 and then infected with IBDV. Confocal and structured illumination microscopy (SIM) analysis showed that SQSTM1 efficiently colocalized with viral dsRNA in IBDV-infected DF-1 cells (Figure 3b,d). To confirm whether IBDV genomic dsRNA directly interacted with the SQSTM1 protein, RNA pull-down was conducted. Prokaryotically expressed recombinant GST-SQSTM1 protein was incubated with purified IBDV dsRNA, followed by GST pull-down. Purified IBDV dsRNA was pulled down by the GST-SQSTM1 protein but not by the GST tag protein (Figure 3e). Furthermore, an RNA pull-down assay was conducted by using biotin-labeled dsRNA as bait. As shown in Figure 3f, the anti-GST antibody recognized an approximately 90 kDa protein in the dsRNA pull-down eluates, while no protein band was observed in the pull-down eluates, suggesting that SQSTM1 binds to dsRNA directly. In addition, anti-SQSTM1 co-immunoprecipitation was performed, and the result showed that endogenous SQSTM1 is bound to IBDV dsRNA. Since dsRNA is bound to SQSTM1 directly, we investigate whether LC3 could colocalize with dsRNA. Confocal microscopy was performed, and results indicated that LC3 colocalized with dsRNA (Figure 3c). In order to determine if SQSTM1 is functioning as a receptor to IBDV dsRNA specifically, two dsRNA nucleic acid analogs, as well as IBDV A and B segments, were transcribed. Nucleic acids analogs were detected on 1% agarose gel (Figure 3h). An RNA pull-down showed that synthetic IBDV A and B segments, but not analog 1 and analog 2, are bound to SQSTM1 directly (Figure 3i). Therefore, SQSTM1 functioned as a receptor for IBDV dsRNA specifically. Furthermore, we next tested the turnover of dsRNA analogs in 293T cells treated with Rapa, an autophagy stimulator. Similarly, the amount of IBDV A and B, but not dsRNA analogs, decreased on treatment with Rapa, suggesting that SQSTM1 selectively degrades IBDV dsRNA (Figure 3j).

### 3.4. SQSTM1 Inhibits IBDV Replication

**Figure 4:**
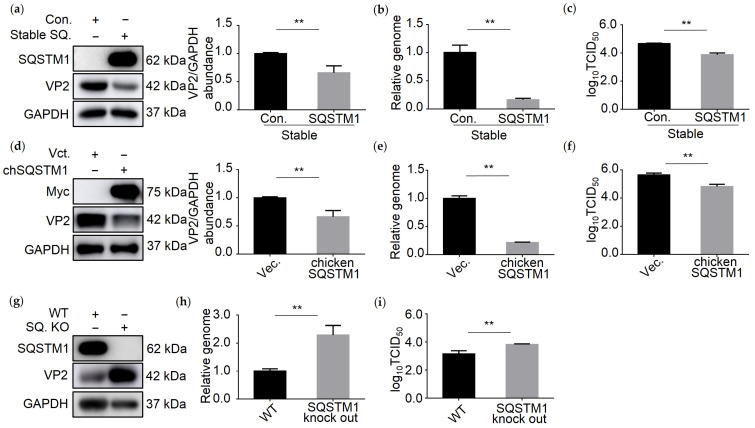
SQSTM1 overexpression, but not knockout, inhibits IBDV replication. (**a**–**c**) Stable expression of SQSTM1 in 293T cells inhibited IBDV replication. STable 293T cells expressing SQSTM1 (Stable SQ.) were infected with IBDV at MOI = 0.1 for 18 h. The cells were collected and equally divided into three parts. IBDV viral protein VP2 was measured by Western blot (**a**). IBDV dsRNA and β-Actin mRNA from 293T cells expressing SQSTM1 were analyzed by qRT-PCR (**b**). The IBDV titer was measured by performing the TCID_50_ assay (**c**). (**d**–**f**) DF-1 cells were transfected with Myc-tag chicken SQSTM1 (chSQSTM1) for 18 h and infected with IBDV at MOI = 0.1 for 18 h. Then, the cells were harvested and equally divided into three parts. One part was subjected to Western blot to quantify the IBDV viral protein VP2 (**d**). Another part was used to extract total RNA, and qRT-PCR was performed to determine the IBDV genome and GAPDH mRNA from DF-1 cells (**e**). The third part was used to determine the IBDV titer by the TCID_50_ assay (**f**). (**g**) SQSTM1 knockout cell lines (*SQ.* KO) were infected with IBDV at MOI = 0.1 for 18 h. The cells were collected and used to measure the abundance of IBDV VP2 by Western blot. (**h**) IBDV dsRNA and β-Actin mRNA from *SQSTM1* KO cell lines were analyzed by qRT-PCR. (**i**) The IBDV titer was tested by performing the TCID_50_ assay. Data were represented as mean ± SD of three independent experiments. NS, not significant (*p* > 0.05); ** *p* < 0.01.

Since SQSTM1 bound to dsRNA directly and promoted the degradation of IBDV dsRNA, we next examined whether SQSTM1 affects IBDV replication. We analyzed the genome level, viral protein VP2, and titer of IBDV in SQSTM1-overexpressing or knockout cell lines infected with IBDV. As shown in Figure 4a, the viral VP2 protein level decreased greatly in 293T cells stably expressing SQSTM1. TCID_50_ and qRT-PCR assays also confirmed that virus titer and genomic copies decreased significantly upon stable expression of SQSTM1 in 293T cells (Figure 4b,c). Similarly, the expression of chSQSTM1 in DF-1 cells also inhibited IBDV infection (Figure 4d–f). Consistent with these findings, *SQSTM1* knockout increased the level of viral proteins, viral genome, and virus titer in 293T cells, further confirming that SQSTM1 inhibited the replication of IBDV (Figure 4g–i). The above results suggest that SQSTM1 is able to inhibit IBDV infection.

### 3.5. R139 and K141 Are Necessary for SQSTM1-Mediated Degradation of dsRNA

**Figure 5:**
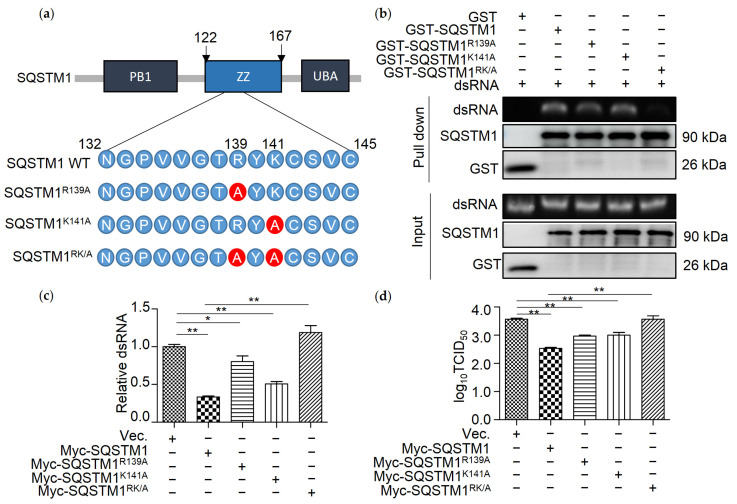
R139 and K141 are necessary for SQSTM1-mediated degradation of dsRNA. (**a**) Diagram of SQSTM1 and its variants SQSTM1^R139A^, SQSTM1^K141A^, SQSTM1^RK/A^. (**b**) R139 and K141 are necessary for dsRNA binding. Prokaryotically expressed protein GST-SQSTM1 and its variants GST-SQSTM1^R139A^, GST-SQSTM1^K141A^, and GST-SQSTM1^RK/A^ were separately incubated with IBDV dsRNA for 4 h, and RNA binding protein immunoprecipitation assay was performed using anti-GST resin. RK/A variant of GST-SQSTM1 showed reduced IBDV dsRNA binding compared to wild type. (**c**) R139 and K141 are critical for degrading dsRNA of SQSTM1. IBDV dsRNA and empty vector (Vct.) or vector expressing Myc-SQSTM1 and its variants (Myc-SQSTM1^R139A^, Myc-SQSTM1^K141A^, Myc-SQSTM1^RK/A^) were cotransfected into 293T cells. Total RNA was extracted, and qRT-PCR was performed to determine the IBDV dsRNA and β-Actin mRNA from 293T cells. (**d**) R139 and K141 are important for the replication of IBDV. Myc-SQSTM1 and its variants (Myc-SQSTM1^R139A^, Myc-SQSTM1^K141A^, Myc-SQSTM1^RK/A^) were transfected into 293T cells, respectively, for 18 h, then infected with IBDV at MOI = 0.1 for 18 h. The replication of IBDV was detected by performing the TCID_50_ assay. Data were represented as mean ± SD of three independent experiments. NS, not significant (*p* > 0.05); * *p* < 0.05, ** *p* < 0.01.

Given that R139 and K141 of SQSTM1 are critical for binding to RNA [5], we supposed that SQSTM1 binds to dsRNA through amino acids R139 and K141. Prokaryotic and eukaryotic vectors expressing single-site mutations of SQSTM1, namely SQSTM1^R139A^ and SQSTM1^K141A^ and double-site mutation, namely SQSTM1^RK/A^, were constructed, respectively (Figure 5a). Prokaryotically expressed recombinant GST-SQSTM1, GST-SQSTM1^R139A^, GST-SQSTM1^K141A^, and GST-SQSTM1^RK/A^ were incubated with purified IBDV dsRNA, respectively, and RNA immunoprecipitation assay was then performed. The results showed that either mutation of R139 or K141 to A slightly, while double mutation of R139 and K141 greatly reduced the affinity of them to IBDV dsRNA compared with WT SQSTM1 (Figure 5b). Next, we wanted to determine whether single or double mutation of R139 and K141 would promote the SQSTM1-mediated degradation of dsRNA. Myc-SQSTM1, Myc-SQSTM1^R139A^, Myc-SQSTM1^K141A^, or Myc-SQSTM1^RK/A^ were separately cotransfected with dsRNA in 293T cells, and the amount of dsRNA was tested by qRT-PCR. As shown in Figure 5c, transfection of Myc-SQSTM1, Myc-SQSTM1^R139A^, Myc-SQSTM1^K141A^, but not Myc-SQSTM1^RK/A^, decreased the level of dsRNA compared to transfection with the empty vector. Furthermore, we wonder if SQSTM1^RK/A^ is able to influence the replication of IBDV. As in Figure 5d, overexpression of Myc-SQSTM1, Myc-SQSTM1^R139A^, Myc-SQSTM1^K141A^ but not Myc-SQSTM1^RK/A^ inhibits the replication of IBDV.

The above results suggest that both amino acid sites K139 and K141 were critical for SQSTM1-mediated dsRNA degradation.

### 3.6. SQSTM1 Is Critical for dsRNA-Induced Production of Interferon β

**Figure 6:**
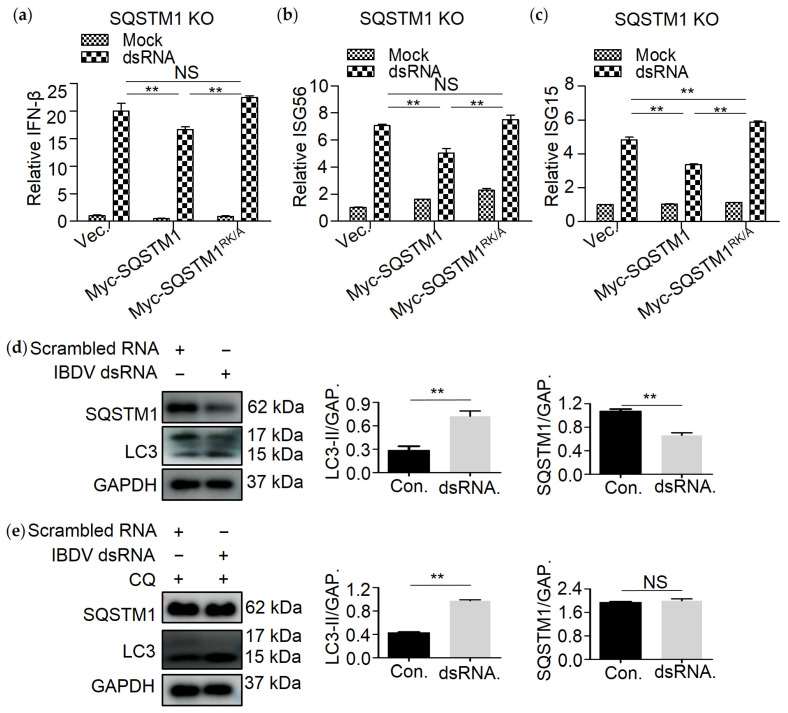
SQSTM1 is critical for dsRNA-induced production of interferon β. (**a**) dsRNA degradation by SQSTM1 inhibited the generation of IFN-β, while its variant SQSTM1^RK/A^ recovered it. SQSTM1 KO cell lines were cotransfected with Myc-SQSTM1 or its variant Myc-SQSTM1^RK/A^ and dsRNA. Cells were collected after 8 h, and RNA was extracted from the cells, and qRT-PCR was conducted to measure the IFN-β and ISG56 (**b**), ISG15 (**c**), and β-Actin mRNA from SQSTM1 KO cell lines. 293T cells were transfected with IBDV dsRNA and treated either with (**e**) or without lysosomal inhibitor CQ (**d**). Scramble RNA was a negative control. Western blot analysis using SQSTM1 and LC3 was performed. Data were represented as mean ± SD of three independent experiments. NS, not significant (*p* > 0.05); ** *p* < 0.01.

Since SQSTM1 mediated degradation of dsRNA, we next tested whether SQSTM1 affects dsRNA-induced production of interferon β (IFN-β). QRT-PCR assay was conducted to detect the mRNA of IFN-β, IFN-stimulated gene 15 (ISG15), or IFN-stimulated gene 56 (ISG56) in dsRNA-treated SQSTM1 KO 293T cell lines. As shown in Figure 6a, cells transfected with dsRNA showed a great increase in IFN-β, suggesting that dsRNA can significantly stimulate the production of IFN-β. While the level of IFN-β, ISG56, and ISG15 decreased greatly upon expression of Myc-SQSTM1, but not Myc-SQSTM1^RK/A^ (Figure 6b,c), suggesting that binding of SQSTM1 to dsRNA is critical for decreasing dsRNA-induced production of IFN-β. To assess an effect of dsRNA on autophagy functions, Western blot analysis using SQSTM1 and LC3 was performed. Compared to scrambled dsRNA, the level of LC3-II increased when transfected with IBDV dsRNA and treated either with or without CQ (Figure 6d,e). In conclusion, IBDV dsRNA can induce autophagy.

## 4. Discussion

The degradation of RNA through a lysosome-dependent pathway was first demonstrated by Sameshima M. et al. in human fibroblasts [20]. As expected, autophagy was then confirmed as a critical mechanism for the degradation of RNA [21,22,23]. Recently, Fujiwara et al. characterized a novel RNA degradation mechanism named “RNautophagy” [24]. Clearance of redundant intracellular RNA is physiologically important. Retrotransposon RNA, such as long interspersed element 1 (LINE 1) and short interspersed nucleotide elements, leads to translocations, inversions, deletions, and amplifications. Mutations in the genome by retrotransposon RNA have been linked to tumorigenesis [25,26]. Guo et al. showed that autophagy plays a critical role in removing retrotransposon RNA and consequently protecting the genome from mutations [9]. Autophagy also plays a significant role in clearing major cellular RNA, namely, rRNA, which is assembled into ribosomes. The autophagic degradation of ribosomes, as well as rRNA, is critical in maintaining cell viability under nutritional stress [27]. Similarly, clearance of stress granules and processing bodies is required because of its physiological importance for cellular viability [28,29]. Our study showed that autophagy is responsible for removing dsRNA of IBDV, confirming the antiviral role of autophagy. However, we could not exclude the possibility that IBDV might employ autophagy to digest naked dsRNA in order to avoid recognition by host immune receptors, thereby preventing the immune response.

Invading viruses and bacteria can be captured by autophagosomes and degraded by lysosomes. Spencer Shelly et al. found that autophagy decreased the replication of VSV, and repression of autophagy led to increased viral replication and pathogenesis in cells and animals [10]. During Sindbis virus (SINV) infection, autophagy is activated and leads to the degradation of viral capsids [11]. Hu et al. found that IBDV could induce autophagy at a very early stage of IBDV infection [30]. Our data indicate that not only viral proteins but also the viral genome can be degraded by autophagy.

Cargo receptors, such as OPTN, NDP52, SQSTM1, and NBR1, mediate selective autophagy. Some of these proteins have been shown to regulate the degradation of nucleic acid. LAMP2C (lysosomal-associated membrane protein 2C) delivers RNA into lysosomes directly for degradation [24]. NDP52 recognizes M. tuberculosis DNA and regulates its autophagic degradation [31]. The SQSTM1 and NDP52 receptors were found to play a role in removing cytoplasmic RNA granules, such as stress granules (SG) and processing bodies (PB) [9]. However, the authors of the study above did not prove the direct binding of RNA to the cargo receptors. In our study, we showed that SQSTM1 was able to bind to dsRNA directly, suggesting that this autophagic cargo receptor might be a cytoplasmic RNA sensor that regulates cytoplasmic RNA degradation. In addition, it is possible that IBDV might employ autophagy to digest naked dsRNA in order to avoid recognition by host immune receptors, thereby blunting the immune response.

In conclusion, we show that SQSTM1 directly binds to IBDV dsRNA and mediates its autophagic degradation, thereby suppressing IBDV replication, highlighting the antiviral role of selective autophagy in IBDV infection.

## Data Availability

Not applicable.

## Supplementary Materials

The following are available online at https://www.mdpi.com/article/10.3390/v13122494/s1, Figure S1: The effects of gene overexpression and compounds on cell viability.
