# Supplementary material for: The Autophagy Cargo Receptor SQSTM1 Inhibits Infectious Bursal Disease Virus Infection through Selective Autophagic Degradation of Double-Stranded Viral RNA
Source: Viruses. 2021 Dec 13;13(12):2494. doi: 10.3390/v13122494 (PMC8704251; doi:10.3390/v13122494)
Supplement: Supplementary file 1 [file viruses-13-02494-s001.zip › viruses-1475729-supplementary.pdf]

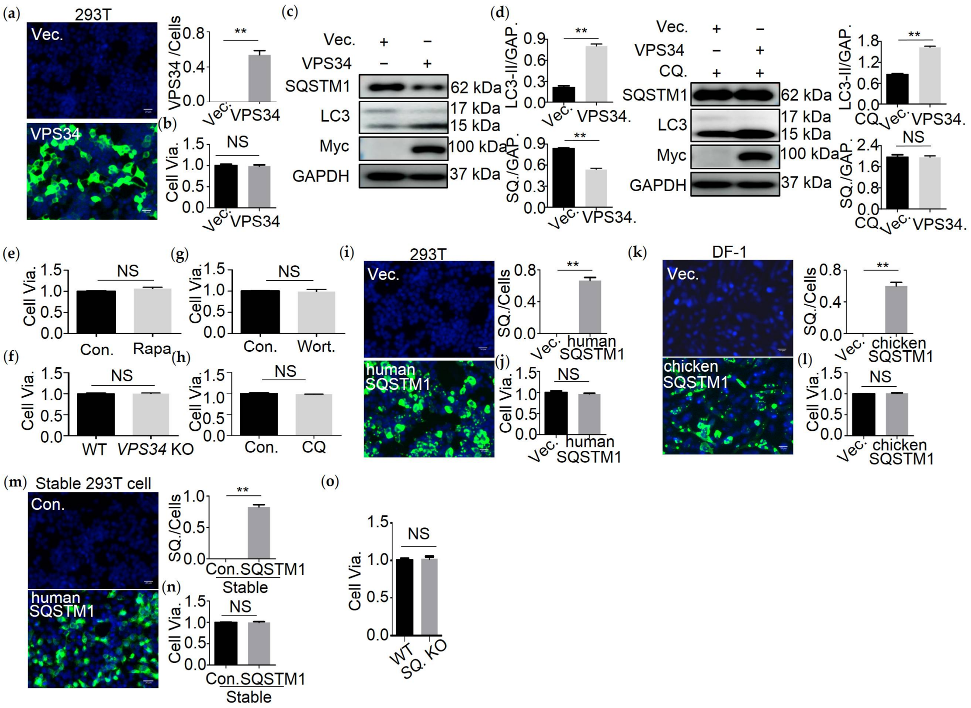

Figure S1. The effects of gene overexpression and compounds on cell viability. (**a**) Cells were transfected with Myc-tag empty vector (Vec.) or Myc-tag human VPS34 (VPS34) and subjected to indirect immunofluorescence assay (IFA) in 293T cells, followed by measuring transfection efficiency. Scale Bar = 20 µm. The cell viability was determined by CCK8 kit in VPS34 overexpressing cells (VPS34) (**b**), VPS34 knockout (VPS34 KO) cell lines (**f**) and cells treated with Rapa (**e**), Wort (**g**), CQ (**h**). (**c**) Quantifications of the immunoblots in cells expressing VPS34. (**d**) Quantifications of the immunoblots in cells expressing VPS34 treated with lysosomal inhibitor CQ. Cells transfected with Myc-tag empty vector (Vec.) or Myc-tag human SQSTM1 (**i**) and chicken SQSTM1 (**k**), or cell lines stable expressing human SQSTM1 (**m**) were subjected to indirect immunofluorescence assay (IFA), followed by measuring transfection efficiency. Scale Bar = 20 µm. The cell viability was determined by CCK8 kit in SQSTM1 overexpressing 293T cells (human SQSTM1) (**j**), chicken SQSTM1 expressing DF-1 cells (**l**), stable 293T cells expressing SQSTM1 (**n**), and SQSTM1 knockout cell lines (**o**).
